# Efficacy and Safety of Inhalation of Nebulized Ethanol in COVID-19 Treatment: A Randomized Clinical Trial

**DOI:** 10.7759/cureus.32218

**Published:** 2022-12-05

**Authors:** Ali Amoushahi, Elham Moazam, Amin Reza Tabatabaei, Golnaz Ghasimi, Ian Grant-Whyte, Pietro Salvatori, Ahmed Ragab Ezz

**Affiliations:** 1 Anesthesiology and Intensive Care Unit, Isfahan University of Medical Sciences, Isfahan, IRN; 2 Cancer Prevention Research Center, Isfahan University of Medical Sciences, Isfahan, IRN; 3 Internal Medicine, Isabn-e-Maryam Hospital, Isfahan University of Medical Sciences, Isfahan, IRN; 4 Family Medicine, Retired, Pointe-Claire, CAN; 5 Otolaryngology, Humanitas San Pio X, Milano, ITA; 6 Anesthesiology, Mansoura University, Mansoura, EGY

**Keywords:** nebulized ethanol, coronavirus disease, coronavirus disease 2019, sars-cov-2, nebulizer, inhalation, ethanol, covid-19

## Abstract

Background: Coronavirus disease 2019 (COVID-19) is a pandemic caused by the SARS-CoV-2 virus. Many efforts have been made and are currently being made to prevent and treat this global disease.

Objectives: This study was designed to evaluate the efficacy and safety of nebulized ethanol (EtOH) in treating COVID-19.

Methods: A randomized clinical trial (RCT) of 99 symptomatic and real-time polymerase chain reaction (RT-PCR)-positive patients admitted to a hospital receiving remdesivir-dexamethasone was conducted. They were randomly assigned to receive distilled water spray (control group (CG)) or 35% EtOH spray (intervention group (IG)). Both groups inhaled three puffs of spray (nebulizer) every six hours for a week. The primary outcome included Global Symptomatic Score (GSS) between the two groups at the first visit and on days three, seven, and 14. Secondary outcomes included the Clinical Status Scale (CSS; a seven-point ordinal scale ranging from death to complete recovery) and readmission rate.

Results: A total of 44 and 55 patients were enrolled in the IG and CG, respectively. Although there was no difference at admission, the GSS and CSS improved significantly in the IG (p = 0.016 and p = 0.001, respectively). The IG readmission rate was considerably lower (0% vs. 10.9%; p = 0.02).

Conclusions: Inhaled-nebulized EtOH is effective in rapidly improving the clinical status and reducing further treatment. Due to its low cost, availability, and absent/tolerable adverse events, it could be recommended as an adjunctive treatment for moderate COVID-19. Further research on curative effects in more serious cases and in prevention is advisable.

## Introduction

The in vitro antiviral effects of ethanol (EtOH) on solving the fat layer [1] and destroying the glycoprotein of coronavirus have already been established [2]. The antiviral effects of EtOH on extracellular surfaces have been demonstrated previously [3]. Immunological studies have shown that acute administration of ethanol can have immunomodulatory effects on the innate immune system mediated by tumor necrosis factor-α (TNF-α) mRNA protein and mitogen-activated protein kinase, and can reduce cytokine storm by reducing inflammatory factors such as toll-like receptor (TLR), toll-like receptor 9 (TLR9), and interleukin-6 [4,5]. It also helps in the chemotaxis of bronchoalveolar macrophages [6]. Other demonstrated effects of ethanol include virus replication by inhibition of RNA-dependent polymerase [7], bronchial dilation by relaxing involuntary smooth muscles [8], sedation and relaxation of the patient [9], and muscular analgesic effects [10]. Ethanol administration has previously been reported to treat methanol poisoning [11], fat embolism [12], prevention of preterm labor [13], preeclampsia [14], and pulmonary edema [15]. The histological safety of inhalation ethanol therapy in the lungs and respiratory tracts of rodents has been demonstrated by Castro-Balado et al. [16]. The Food and Drug Administration approved ethanol. Cytokine storms cause many deaths due to the coronavirus disease 2019 (COVID-19) [17]. Given the actions of ethanol on virus wall destruction, inhibition of proliferation, and inhibition of immune hyperactivity, the question now is, "Can ethanol inhalation therapy be effective in controlling COVID-19?" There is no knowledge about inhaled ethanol therapy for COVID-19. This idea was first suggested and published one month after Iran's COVID-19 pandemic (February 2020) [18,19]. Later, a study dealing with the rationale for ethanol use in this field was presented [20]. A randomized clinical trial (RCT) evaluating the combined administration of dimethyl sulfoxide and ethanol recently showed considerable COVID-19 prevention in healthcare providers [21]. To try to find the answer, we conducted a randomized clinical trial aimed at evaluating the effectiveness of ethanol therapy on the clinical status and prognosis of a defined set of patients. The study was approved by the Research and Ethics Committee of the School of Medicine, Isfahan University of Medical Sciences, and registered at the Iranian Registry of Clinical Trials (https://irct.ir/trial/58201).

This article was previously posted to the medRxiv preprint server on June 23, 2022.

## Materials and methods

Study design and oversight

**Figure 1:**
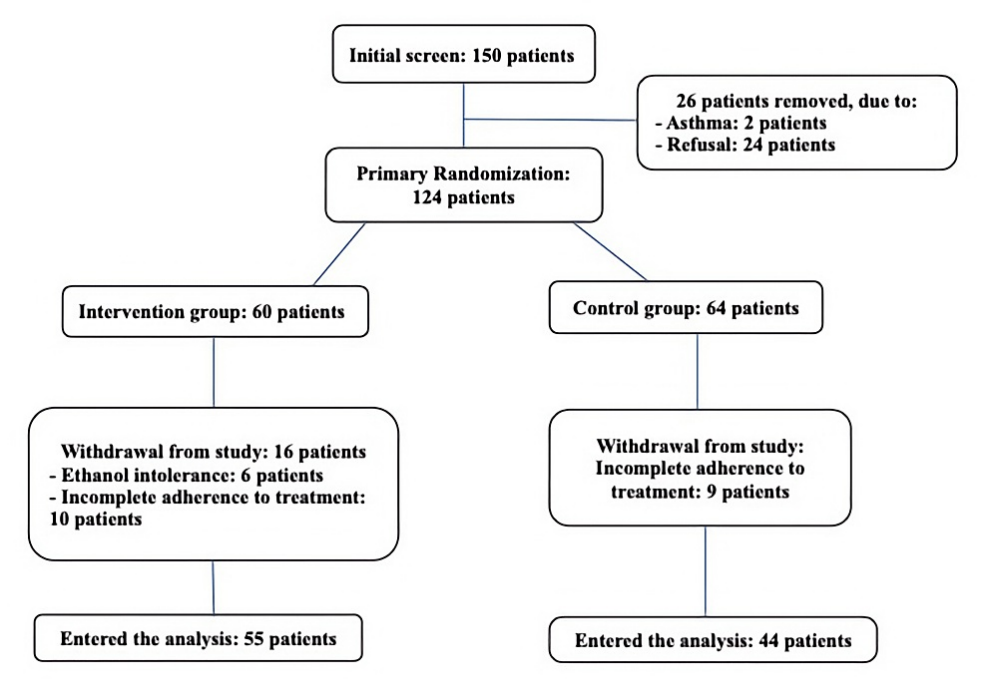
Study flowchart

This study is a randomized, double-blind, clinical trial with a control group and parallel design that was conducted at the Isabn-e-Maryam Hospital at the Medical University of Isfahan, Iran, in September 2021 (Figure 1). The patients were randomly assigned in a 1:1 ratio. The study was originally intended for patients admitted to the hospital; however, due to amending the country's policy of setting up respiratory clinics in hospitals and prescribing remdesivir and dexamethasone to patients with moderate COVID-19, the study was conducted in this clinic. Physicians of hospitalized patients were informed about the patient's referral to the respiratory clinic and this study.

Patients

The study population consisted of polymerase chain reaction (PCR)-positive SARS-CoV-2 patients. They had moderate COVID-19 based on the national guideline for COVID-19 management, i.e., oxygen saturation = 90-94% or lung involvement [22], referred to the respiratory clinic of the hospital. Inclusion criteria were as follows: agreeing to implement the plan in the form of informed consent, age over 12 years, no pregnancy, no history of asthma, alcoholism, or epilepsy, no contraindications to ethanol, and use of drugs that interact with ethanol. Exclusion criteria were intolerance to inhaled ethanol and partial or incomplete treatment. An ethanol patch skin test was used to detect possible alcohol allergies. In this experiment, a drop of ethanol was placed on a gauze pad and attached to the patient's arm. After approximately seven minutes, symptoms, such as redness, swelling, or itching of the skin, were observed. These symptoms indicate the possibility of allergy or intolerance to alcohol.

Intervention

Both the control group (CG) and intervention group (IG) were enrolled in the standard treatments based on the national clinical guidelines of Iran [21]. The national standard treatment included intramuscular dexamethasone 8 mg/day for five days, and 200 mg of remdesivir intravenously on day one, followed by 100 mg of remdesivir once daily for four days, infused over 30-60 minutes. In addition to the standard treatment, patients were randomly assigned to either the CG (distilled water spray) or the IG (35% ethanol spray). Two sets of 100 ml spray were provided and delivered according to randomization. All patients were instructed to spray the mask three times a day (every six to eight hours) and take deep breaths. We emphasized that this protocol had to be repeated for seven days, depending on the persistence of symptoms. Patients were taught by nurses until they were able to manage the procedure and were then allowed to do it on their own. Patient compliance was assessed at all referrals and follow-up appointments. Failure to adhere to the protocol (spray not used or irregularly used) resulted in withdrawal from the study.

Clinical and laboratory monitoring

The data collection sheet consisted of two sections: demographic and clinical information. Demographic information was obtained from the patient's records and the data collection checklist was completed by a trained nurse based on clinical symptoms, clinical results, and clinical examination. Data related to research variables, including blood oxygen saturation in pulse oximetry, need for adjunctive treatment or readmission in the hospital, and clinical symptoms in both groups, were collected until discharge.

Study outcomes

Primary Outcomes

The Global Symptomatic Score (GSS) is considered an indicator of the clinical status of patients and is achieved by calculating the cumulative scores of clinical signs and symptoms, including fever, headache, body aches, sore throat, runny nose, chills, cough, shortness of breath, anorexia, loss of smell, and loss of taste. This index was designed to summarize clinical symptoms in the form of one index.

Oxygenation status was monitored and recorded daily using a pulse oximeter. The pulse oximeter was fixed, and the patient was breathing room air at the time of measurement without supplemental oxygen. Inflammatory status was defined as the variation in serum C-reactive protein (CRP) levels.

Secondary Outcomes

A modified seven-point ordinal scale was used to assess clinical conditions on day 14 of the treatment period related to research [23]. This scale includes seven indices: (1) death; (2) hospitalized, on invasive mechanical ventilation; (3) hospitalized, non-invasive ventilation or high-flow oxygen devices; (4) hospitalized, requiring low-flow supplemental oxygen; (5) hospitalized, for whatever reason, requiring ongoing medical care (related to COVID-19 or otherwise), requiring supplemental oxygen at home; (6) continued signs or symptoms of COVID-19 without requiring supplemental oxygen, no longer require ongoing medical care; (7) complete recovery and any possible side effects were reported and treated in both groups. The need for hospitalization in the intensive care unit, drug side effects, clinical symptoms, and mortality of the research samples were recorded and monitored in both groups. The final follow-up was performed on the 14th day of the disease. The last follow-up was performed on the 14th day of the disease. During follow-up, physical examinations, history, telephone calls, review of patient records, and hospital information system documents were used. Side effects were recorded after obtaining informed consent. We have made some changes to the primary endpoints. This was because of the limitations that occurred at the same time as the peak of the disease in Iran with the implementation of this study, and patients who needed to be hospitalized were followed up on an outpatient basis in the respiratory clinic. We provided a detailed account of the changes in the protocol to the sponsor and the institutional review board. The main predicted outcome was the length of stay. During the surge, all moderate patients were treated on a five-day schedule; therefore, this index was replaced with a more comprehensive clinical status.

Sampling

Sampling was performed using an easy random sampling method. A computerized random number table was used for random assignment. One nurse determined the sequence of random allocations. She kept the randomization list private and allocated each person if they were eligible and consented to the trial. Another nurse filled the sprays (nebulizer) one by one with 100 ml of diluted distilled water or ethanol (35%) and labeled them with the numbers coming out of the list one by one. Each spray was delivered to one of the participants, and their family or companion was instructed on how to use it. Clinicians, nurses, and analysts performed blinding.

Statistical analysis

We calculated that 88 patients (44 in each group) would provide greater than 90% power to detect an odds ratio of 3 for the ethanol group versus the placebo group using a two-sided significance level of 0.05. Based on the "treatment-on" or "per-protocol" strategy, analysis was limited to participants who, according to the study protocol and inclusion criteria, received full interventions and informed the outcomes. Quantitative and qualitative variables were reported as descriptive statistics, including means, standard deviations, and numbers (%). Qualitative variables were compared between the two groups using the chi-square test, and comparisons between oxygen saturation (SpO2) values and GSS on days one, three, seven, and 14 were performed using a mixed model. The mean changes in baseline values were measured using repeated-measures analysis. The sphericity hypothesis was rejected with the help of Mauchly's statistics, and the Geisser-Greenhouse correction was used for this purpose. The proportion of patients who needed additional medical care after 14 days was tested in the two groups using the χ2 test, which was performed at zero, three, seven, and 14 days after the intervention, and cumulative odds ordinal logistic regression with proportional odds was used to compare clinical status between the two groups on day 14. An odds ratio greater than 1 indicated changes in clinical status across all categories toward category seven for the intervention group vs. the standard care group. For clinical status, if a patient recovered, the ordinal score was recorded as 7 on the day of recovery and all subsequent days unless the patient was hospitalized for COVID-19-related reasons or others. All statistical analyses were performed using SPSS version 22 (IBM Corp., Armonk, NY), and p < 0.05 was considered significant. The patient's gender variable was controlled in the outcome indicators.

## Results

Patient characteristics

In this study, 150 patients from the COVID-19 respiratory outpatient clinic of Isabn-e-Maryam Hospital of Isfahan University of Medical Sciences were evaluated for inclusion based on the positive result of the real-time polymerase chain reaction (RT-PCR) test, from September to November 2021. Two patients did not meet the inclusion criteria and 24 patients did not agree with the study. The remaining 124 patients were randomly assigned into two groups (intervention and control). Twenty-five patients were then excluded from the study, due to intolerance to ethanol inhalation (six patients); hiccups, eye irritation, cough, shortness of breath, sneezing, and the unpleasant smell of alcohol were the main reasons for their intolerance. On the other hand, 19 patients (10 in the IG and nine in the CG) were excluded from the study due to irregularity or not following the recommended method. Finally, 99 patients entered the analysis: 44 patients in the IG and 55 patients in the CG.

Table 1 shows the demographic and baseline characteristics of patients in the two groups. The male/female ratio was 43/56 patients (42.4/56.6%). The mean age of patients was 46.4 years. Thirty-eight patients had comorbidities. The most common underlying disease in both groups was diabetes mellitus: four (7%) in the CG and six (14.3%) in the IG. Seven patients had hypertension and seven patients had other cardiovascular problems. The two groups did not have significant differences in terms of mean age, weight, level of education, and the number of risk factors.

**Table 1:** Demographics

	Intervention group (N = 44, 44.4%)	Control group (N = 55, 55.6%)
Age (years), mean (±SD)	45.91 (±12.58)	46.15 (±13.15)
BMI, N (%)		
Normal weight	9 (20.5)	16 (29.1)
Overweight	22 (50)	25 (45.5)
Obesity	10 (22.7)	10 (20)
Excessive obesity	3 (6.8)	3 (5.5)
Gender, N (%)		
Female	19 (43.2)	37 (67.3)
Male	25 (56.8)	18 (32.7)
Education level, N (%)		
Illiterate and elementary	8 (16.3)	3 (5.4)
Secondary	7 (14.3)	8 (14.3)
Diploma	16 (32.7)	28 (50)
Bachelor-higher	16 (30.6)	16 (26.8)
Unknown	3 (6.1)	2 (3.6)
Risk factors for disease, N (%)		
None	38 (69.1)	23 (52.3)
1 risk factor	12 (21.8)	19 (43.2)
2 risk factors	4 (7.3)	2 (4.5)
3 risk factors	1 (1.8)	0

Clinical signs and symptoms at the time of admission

Patients also did not differ significantly in the time distance between the onset of symptoms and admission, pulmonary involvement, and early clinical signs and symptoms at baseline. The basic characteristics of the patient's clinical signs and symptoms are given in Table 2.

**Table 2:** Baseline signs and symptoms, risk factors, and laboratory values

	Intervention group (N = 44, 44.4%)	Control group (N = 55, 55.6%)
Delay from symptoms onset to treatment, mean (±SD)	8.50 (±3.52)	9.36 (±5.13)
Pulmonary involvement (CT scan)		
Less than 30%	19 (43.2)	22 (40)
30-49%	15 (34.1)	25 (45.5)
50% or more	2 (4.5)	2 (3.6)
Unknown	8 (18.2)	6 (10.9)
Fever	25 (56.8)	21 (38.2)
Chills	30 (68.2)	35 (63.6)
Cough	41 (95.3)	49 (89.1)
Headache	30 (68.2)	35 (63.6)
Short breath	24 (54.5)	37 (67.3)
Sore throat	16 (36.4)	27 (49)
Rhinorrhea	9 (20.5)	18 (32.7)
Body pain	36 (65.5)	36 (65.5)
Anorexia	29 (65.9)	38 (69.1)
Anosmia	26 (59.1)	39 (70.9)
Lack of taste	27 (61.4)	32 (58.2)
Global Symptomatic Score (GSS)	6.72 (2.07)	6.67 (2.09)

The primary clinical symptoms in the IG were cough, body aches, chills, and headaches. Cough, olfactory disturbance, and anorexia were more common in the CG. No significant difference was observed in symptoms.

Global symptomatic score

**Figure 2:**
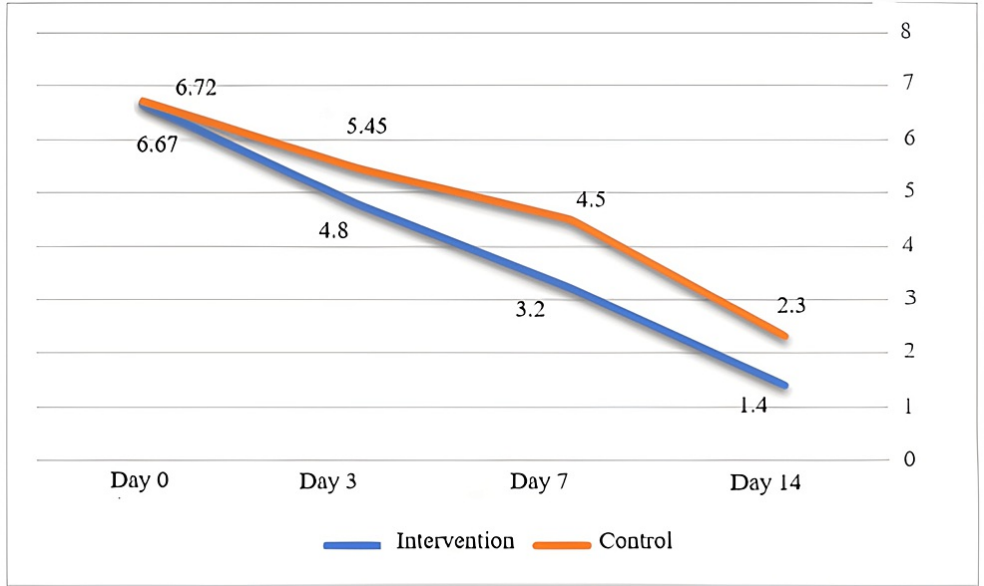
Comparison of mean Global Symptomatic Score (GSS) between the intervention and the control groups at admission, and days three, seven, and 14 after admission

The GSS at the beginning of treatment and after three, seven, and 14 days of treatment was assessed in two groups. The results are shown in Figure 2.

Statistical analysis showed that the GSS of the two groups was the same at the beginning of the study, but in the IG, the clinical symptoms decreased more than in the placebo group and more rapidly. This difference was statistically significant (p = 0.016).

Blood oxygen saturation

**Figure 3:**
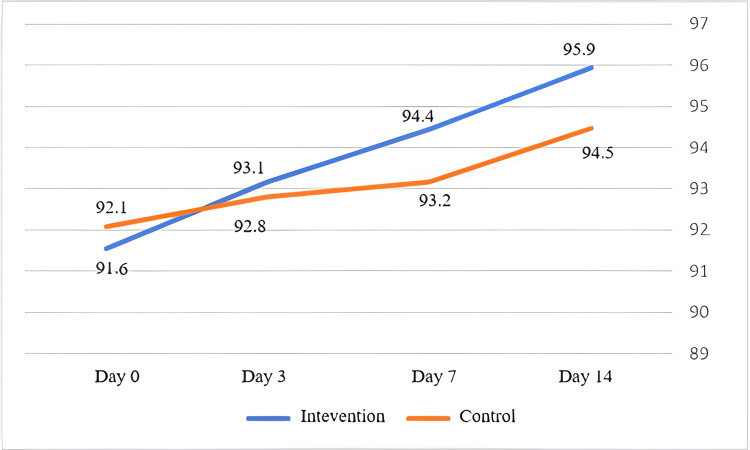
Comparison of mean blood oxygen saturation (SpO2) between the intervention and the control groups at admission, and days three, seven, and 14 after patient admission

There was no significant difference in blood oxygen saturation between the two groups at the time of the study (92.07 ± 4.6 in the CG vs. 91.56 ± 3.39 in the IG). As can be seen in Figure 3, blood oxygenation improved in both groups and the slope of oxygenation was higher in the ethanol group. Although, this difference is not statistically significant (p = 0.097).

Inflammatory factor (CRP)

**Figure 4:**
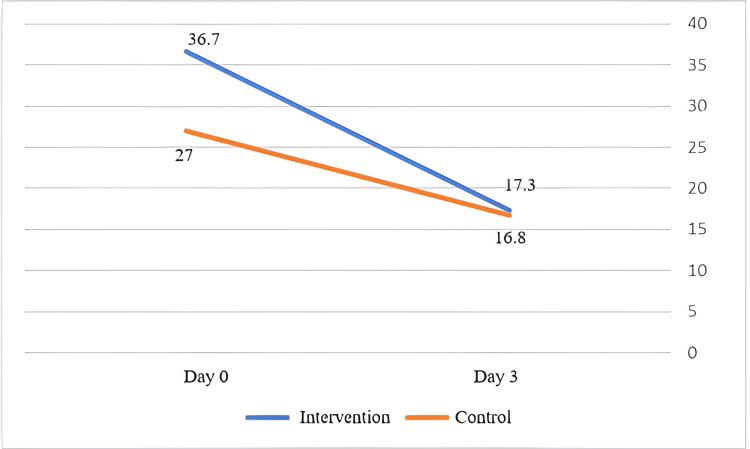
Comparison of C-reactive protein between the intervention and control groups at admission and three days after patient admission

Multiple measurements and statistical analysis of the two groups showed that CRP had a decreasing trend (Figure 4), but in the IG, the rate of reduction was more rapid and intense, in a significant way (p = 0.05).

Clinical status scale

CSS based on the modified seven-point ordinal scale was compared in two groups. On day 14, the odds of the IG having better CSS were 5.7 times higher (95% CI: 2.47-13.19) than that of the CG, with a statistically significant effect: Wald χ2 (1) = 16.67 and p = 0.001. Details are shown in Table 3.

**Table 3:** Clinical Status Scale (CSS) on day 14 from admission

Characteristic and score, N (%)	Intervention (N = 44)	Control (N = 55)
Death	0	0
Hospitalized, on invasive mechanical ventilation	0	0
Hospitalized, on non-invasive ventilation or high-flow oxygen devices	0	0
Hospitalizations for any reason and need oxygen	0	2 (3.63)
Requiring ongoing medical care or supplemental oxygen at home	2 (4.54)	10 (18.18)
Continue signs or symptoms without requiring supplemental oxygen - no longer requires ongoing medical care	13 (29.54)	29 (52.72)
Complete recovery	29 (65.90)	14 (25.45)

After the end of the treatment period in the CG, six patients (10.9%) were readmitted to receive additional treatment or hospitalization. In the ethanol group, none of the patients were readmitted (p = 0.02).

Adverse events and safety

Six out of 50 patients in the ethanol group (12%) stopped using it because of adverse events at the onset of inhalation and we excluded them from the study. No more than one case was observed for each side effect and that side effect disappeared after stopping ethanol use. Adverse events included hiccups, eye irritation, cough, shortness of breath, sneezing, and an unpleasant odor of alcohol.

## Discussion

In this clinical trial, patients with positive RT-PCR test, moderate clinical symptoms, and suitable for remdesivir and dexamethasone treatment (according to the Iran Ministry of Health protocol) were included and the effect of adding nebulized inhalation was studied. The rationale for using EtOH in COVID-19 has been extensively treated [20].

Experimental and clinical data leave no doubt about the effect of ethanol on destroying or inactivating SARS-CoV-2, even at concentrations as low as 30% v/v and for a short time (30 seconds) [24]. The EtOH viricidal properties rely on the dissolution of the virus fat layer and subsequent inhibition of its proliferation. In addition, EtOH has been shown to mitigate the hyperactivity of the immune system during COVID-19. Ethanol is likely ineffective against intracellular viruses.

Since viral replication occurs in 48-72 hours, followed by cellular death and shedding, it is important to prolong ethanol inhalation for at least three days. Moreover, thanks to its non-specificity, ethanol is intrinsically effective on any SARS-CoV-2 variant and other “enveloped” viruses.

Worse outcomes in ICU patients were related to the abnormal presence of *Mycoplasma salivarium* in the lower tract or *Clostridia* absence in the upper tract [25]. Interestingly, it should be noted that *Mycoplasma* and SARS-CoV-2 are completely inactivated by ethanol [26]. Moreover, certain *Clostridia* strains produce endogenous ethanol [27]. Hypothetically, the absence of nasopharyngeal *Clostridia* could lead to a lack of local ethanol production and therefore reduced/absent inactivation of SARS-CoV-2 at this level, thus allowing the virus to spread to the lower respiratory tract.

Concerns about the mucosal damage that inhaled ethanol could induce locally have been frequently and strongly raised. The meticulous work of Castro-Balado et al. [16] seems to have definitively eliminated these concerns.

When evaluating methods, it should be highlighted that spraying into the mask reduces the nebulized liquid's dispersion and evaporation, leading to a longer-lasting action, and maintaining its efficacy. A possible unavoidable bias is derived from the two solutions' distinct odors. However, as the patients did not know the real substance in the spray, they could only be aware that one spray was different from the other. In other words, the drug could have been in an odorless spray. The COVID-19 standard treatment includes intramuscular dexamethasone and remdesivir [22]. A recent study has shown no benefit on the 30-day mortality from adding early antibiotic treatment for COVID-19 pneumonia [28]. Contrary to expectations [29,30], the authors [28] did not find significant vaccine effectiveness in lowering disease severity and mortality among patients with COVID-19 pneumonia.

Our results showed that in the IG, the GSS decreased more than in the control group, and these data reached a statistically significant level (p = 0.016).

The benefits of nebulized EtOH inhalation were also significantly evident (p = 0.05) in reducing the inflammatory status, as measured by CRP. This finding confirms the beneficial effects of EtOH on the immune system [3].

On the other hand, in the IG, blood oxygenation improved faster, and the slope of oxygen was higher than that in the CG. However, there was no statistically significant difference between the two groups (p = 0.097).

About CSS, the response of the IG was better than that of the CG because no patient was readmitted, against the need to repeat the standard treatment or to be hospitalized, which occurred in six patients (10.8%) in the CG. These findings support the EtOH virucidal power.

One of the limitations of this trial was that the patients were far from our strict control and switched to other therapies. In addition, unpleasant alcohol consumption and the possibility of non-inhaled alcohol consumption are limiting concerns of the healthcare system. Among others, this study is limited by confounding factors (due to an imbalance in gender distribution), chance (due to the small sample size), and insufficient power. A per-protocol analysis breaks the randomization sequence and introduces bias into the study.

## Conclusions

All over, adding inhaled nebulized ethanol to the standard treatment (remdesivir + dexamethasone) significantly improves recovery from moderate COVID-19. Considering the low cost, availability, and lack of significant adverse events associated with ethanol, it could be recommended as an adjunctive treatment for moderate COVID-19. Further research on curative effects in more serious cases and in prevention is advisable.
